# The molecular mechanisms of cuproptosis and its relevance to atherosclerosis

**DOI:** 10.17305/bb.2024.11826

**Published:** 2025-01-15

**Authors:** Jiankang Wang, Zhian Chen, Hang Shang, Jiajuan Guo

**Affiliations:** 1 College of Traditional Chinese Medicine, Changchun University of Traditional Chinese Medicine, Changchun, China; 2 Department of Cardiovascular Medicine, Affiliated Hospital of Changchun University of Traditional Chinese Medicine, Changchun, China

**Keywords:** Cuproptosis, atherosclerosis, AS, copper, tricarboxylic acid, TCA cycle, reactive oxygen species, ROS

## Abstract

Atherosclerosis (AS) is a chronic inflammatory disease associated with lipid deposition in the vascular intima. Copper is a vital trace element implicated in the onset and progression of AS. Excessive intracellular copper accumulation induces a unique form of cell death termed “cuproptosis.” The emergence of the concept of cuproptosis has highlighted the potential role of copper in AS. This review explores the regulatory mechanisms of copper metabolism and cuproptosis, summarizes recent findings on the link between copper excess and AS, and examines how cuproptosis may influence AS progression. The goal is to propose novel diagnostic and therapeutic strategies for AS through the lens of cuproptosis.

## Introduction

Atherosclerosis (AS) is a chronic inflammatory condition characterized by the accumulation of lipids, inflammatory cells, and extracellular matrix within arterial walls, leading to the formation of atherosclerotic plaques [1, 2]. Over the past two decades, cardiovascular diseases (CVDs) have remained the leading global cause of morbidity and mortality [3]. AS is the primary cause and pathological foundation of CVDs [4] and presents a significant challenge in their prevention and treatment [5]. Arterial stenosis and plaque rupture caused by AS can result in severe cardiovascular and cerebrovascular events, including myocardial infarction, coronary artery disease, and stroke. Globally, AS is responsible for approximately 20 million deaths annually, with its prevalence rising among younger populations—posing a serious threat to public health and life expectancy [6]. Consequently, identifying effective strategies to prevent or mitigate AS has become a critical focus of medical research. The pathophysiological mechanisms underlying AS are highly complex, involving processes, such as inflammation, oxidative stress, and lipid metabolism [7–9]. Despite significant progress in AS research, its exact pathogenesis remains poorly understood, underscoring the need for further exploration to identify novel therapeutic targets [10]. Copper (Cu) plays a critical role as a catalytic cofactor in various physiological processes, including energy metabolism, mitochondrial respiration, and antioxidation [11]. Both excess and deficiency of copper can lead to pathological changes that negatively impact human health [12]. Research has shown that copper promotes endothelial cell (EC) proliferation and migration, processes integral to angiogenesis [13]. Under normal conditions, copper helps maintain the structure and function of blood vessels. In cases of vascular injury, copper enhances EC and fibroblast proliferation and differentiation by upregulating VEGF, FGF, and other related factors, thereby protecting cells from hypoxic damage [14, 15]. However, elevated serum copper levels have been associated with AS and other inflammatory vascular diseases, correlating positively with an increased risk of coronary heart disease (CHD) [16, 17]. Epidemiological studies further support the link between high serum copper levels and a greater risk of atherosclerotic diseases [18, 19]. Maintaining copper homeostasis is essential for preserving vascular integrity and preventing AS, as excessive copper accumulation can damage blood vessels.

Tsvetkov et al. recently identified a novel copper-dependent cell death pathway, known as cuproptosis, which differs from oxidative stress-induced cell death mechanisms, such as apoptosis, ferroptosis, and necrosis. Cuproptosis is closely linked to mitochondrial respiration [12]. This process involves copper binding to fatty acylated proteins within the tricarboxylic acid (TCA) cycle, resulting in protein aggregation, a reduction in Fe–S cluster proteins, and subsequent protein toxicity, ultimately leading to cell death. Cuproptosis has been implicated in various diseases, including Wilson’s disease, a rare autosomal recessive disorder of copper metabolism. Currently, copper and ATP7B are considered critical therapeutic targets for managing Wilson’s disease. Interestingly, in patients with Wilson’s disease, cuproptosis is often accompanied by ferroptosis [20, 21]. Clinical research and animal studies suggest that disruptions in serum copper homeostasis and cuproptosis are associated with the onset and progression of CVDs [19, 22]. Antioxidants may slow CVD progression by inhibiting cuproptosis [23, 24]. Moreover, cuproptosis can induce cell death and mitochondrial dysfunction, which can accelerate the progression of AS [25, 26]. However, the precise mechanisms through which cuproptosis contributes to AS remain unclear. This review seeks to summarize current knowledge on copper metabolism and the mechanisms underlying cuproptosis, explore its potential role in AS, and identify related diagnostic genes and therapeutic strategies. By doing so, this review aims to provide a comprehensive understanding of cuproptosis in AS and uncover novel diagnostic and therapeutic targets for its management.

## Biological characteristics and metabolic process of copper

### Biological characteristics of copper

Copper is a trace metal element in the human body with redox activity, playing a dual role in supporting key metabolic enzymes and generating reactive oxygen species (ROS) [27]. Beyond its redox function, copper is essential for embryonic development, hemoglobin regulation, and the proper functioning of liver cells and neurons. The total copper content in the human body is approximately 100 mg [28], with a recommended daily intake of 0.9 mg for healthy adults. Copper is primarily absorbed in the small intestine via amino acid transporters, with an absorption efficiency of around 40%. Tissue copper concentrations range from 1 to 10 mg/g, while plasma levels are approximately 1000 ng/mL [29]. Dietary copper, primarily present as Cu^2^^+^, is absorbed by intestinal epithelial cells, stored in the liver, and excreted via the gallbladder. Copper ions exist in two oxidation states—Cu^+^ and Cu^2^^+^—with interconversion mechanisms that maintain copper homeostasis [30]. In the duodenum and small intestine, Cu^2^^+^ is reduced to Cu^+^ by metal reductases. The reduced Cu^+^ then binds to copper transporters, such as ceruloplasmin (CP), albumin, and other trans-copper proteins, enabling transport to various organs and tissues [28]. Once in the bloodstream, approximately 70% of copper binds to CP, 15% to albumin, and 10% to α-2 macroglobulin [31].

### Cellular copper homeostasis

**Figure 1.**
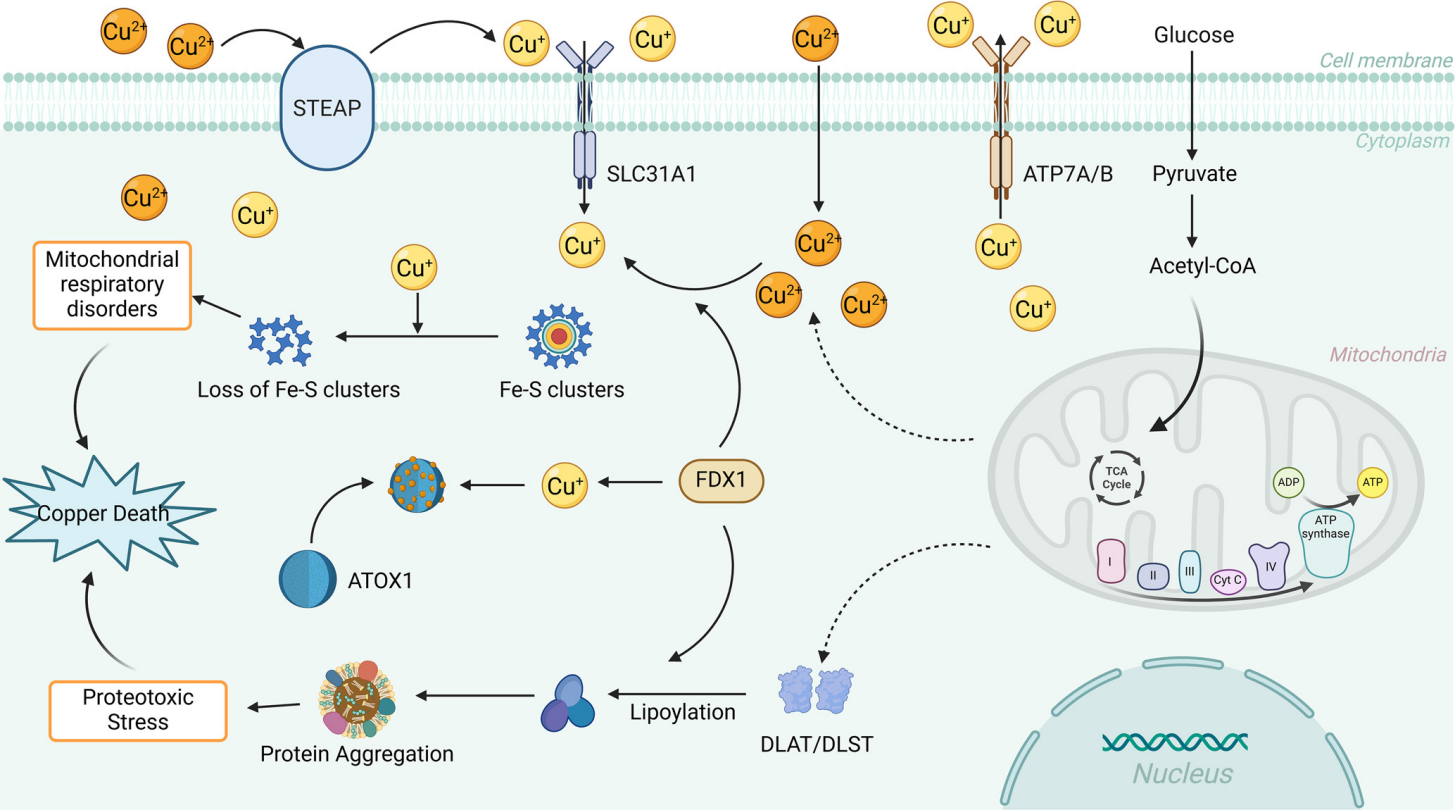
**Copper metabolism pathway and cuproptosis regulation mechanism.** Cu^2+^ is reduced to Cu^+^ on the surface of digestive tract epithelial cells by STEAP, and then enters the cells through high affinity transmembrane transporter CTR1. Cu^+^ will bind to copper chaperones such as COX17, CCS and ATOX1 through cytoplasm, mitochondria and Golgi apparatus, and deliver them to different copper proteins in a targeted way to maintain the stability of copper ions. Excessive or lack of copper ions will induce the occurrence and development of copper death through mitochondrial dysfunction and protein toxic reaction. FDX1: Ferredoxin 1; ATOX1: Antioxidant copper chaperone 1; TCA: Tricarboxylic acid; Cyt C: Cytochrome C.

Cu^2^^+^ is reduced to Cu^+^ on the surface of digestive tract epithelial cells by the metal reductase STEAP and subsequently enters these cells via the high-affinity transmembrane transporter, copper transporter 1 (CTR1) [32]. Once inside the cell, Cu^+^ binds to copper chaperones located in the cytoplasm, mitochondria, and Golgi apparatus, ensuring its delivery to specific copper-dependent proteins. The primary copper chaperone proteins identified to date include cytochrome c oxidase copper chaperone 17 (COX17), superoxide dismutase copper chaperone (CCS), and antioxidant copper chaperone 1 (ATOX1) (Figure 1). COX17 is predominantly localized in the cytoplasm and mitochondrial membrane. In fungi, COX17 has been shown to exhibit cytochrome oxidase activity [33], whereas in mammals, its primary function is to activate cytochrome c oxidase (CCO) and support embryonic development [34]. COX17 binds Cu^+^ and transfers it to secondary copper-binding proteins, such as SCO1/2 and CCO, to enhance enzymatic activity within the respiratory chain [35]. The CCS delivers Cu^+^ to superoxide dismutase 1 (SOD1), enabling its antioxidative stress function [36]. Studies have demonstrated that SOD1 activity decreases while its sensitivity to stress increases in CCS gene knockout mice, confirming that CCS is the primary pathway by which SOD1 acquires Cu^2^^+^ [37]. ATOX1 transports Cu^+^ to the nucleus, where it interacts with transcription factors to regulate gene expression. Additionally, ATOX1 facilitates the transfer of Cu^+^ from the trans-Golgi network (TGN) to copper transporter ATPase. Under normal physiological Cu^+^ concentrations, copper transporter ATPase is localized in the TGN region. However, when intracellular Cu^+^ levels exceed physiological thresholds, copper transporter ATPase relocates to the cell membrane to expel Cu^+^ via energy-dependent transport mechanisms [38, 39]. Unbound free copper interacts with mitochondrial proteins and participates in the Fenton reaction, which generates ROS and triggers cell death mediated by protein toxicity stress [12]. Copper is essential for cellular function and plays a critical role in numerous physiological processes. However, both excess and deficiency of copper ions can lead to cytotoxicity and cell death through various mechanisms, underscoring the importance of maintaining copper homeostasis.

## The research progress and mechanism of cuproptosis

### Research progress of cuproptosis

The phenomenon of copper-induced cell death was first identified in the early 1980s [40]. In 1978, researchers discovered the mechanism regulating intracellular copper levels in normal fibroblasts and observed that excessive copper concentrations led to cell death. Fibroblasts exposed to copper concentrations exceeding 30 µg/mL in the culture medium experienced a 19-fold increase in intracellular copper levels compared to baseline conditions, ultimately resulting in cell death [41]. Elevated copper levels drive the production of ROS, which lead to oxidative stress, DNA damage, and, eventually, cell death. These findings spurred further research into the molecular mechanisms underlying copper-induced cell death and its role in disease processes. Notably, elevated copper levels induce not only oxidative stress and DNA damage [42] but also trigger cell death through apoptosis or caspase-independent pathways. Over time, researchers have elucidated the complex interplay between copper ions, oxidative stress, apoptosis, and disease progression [43, 44], highlighting the importance of understanding copper-induced cell death. In March 2022, Tsvetkov et al. [12] published groundbreaking research on the mechanism of copper-induced cell death, which they termed cuproptosis. Cuproptosis is distinct from other forms of cell death, characterized by abnormal aggregation of acylated proteins and depletion of iron-sulfur cluster proteins, resulting in morphological changes, such as mitochondrial contraction, chromatin rupture, and cell membrane damage [45]. The TCA cycle plays a central role in cuproptosis. Tsvetkov et al. [12] identified the copper ion carrier Elisimo through their experiments and demonstrated several regulatory mechanisms influencing cuproptosis. Notably, they showed that mitochondrial-respiring cells are particularly sensitive to copper-loaded Elisimo. Upon entry into mitochondria, copper ions do not directly target the electron transport chain; rather, they induce oligomerization of acylated proteins, regulated by Ferredoxin 1 (FDX1). This process drives key enzymes into the TCA cycle, disrupting metabolic processes and triggering cell death [46].

### Mechanism of cuproptosis

#### Copper induces oxidative stress injury

Oxidative stress arises from an imbalance between oxidative and antioxidative processes in the body, with mitochondria being the primary site of oxidative damage. This imbalance causes irreversible harm to mitochondria and cellular proteins, leading to the release of mitochondrial cytochrome C (Cyt C) and activation of the mitochondrial apoptosis pathway [47]. Rakshit et al. [48] demonstrated that copper induces oxidative stress, ultimately triggering apoptosis. For instance, Cu^+^ in hepatocytes contributes to the synthesis of SOD1 through its interaction with copper peroxide partners, maintaining the stability of the antioxidant enzyme system [49]. The continuous accumulation of ROS at the substrate end of the mitochondrial respiratory chain is a critical factor in oxidative stress. Mitochondria are both the main producers and primary targets of excessive ROS, which promote apoptosis. The mitochondrial apoptosis pathway plays a central role in cuproptosis. Upon entering hepatocytes, a portion of copper is transported to mitochondria, where it activates CCO [50]. Excessive copper exposure disrupts antioxidant enzyme activity, induces ROS overproduction, impairs mitochondrial electron transport, and activates the mitochondrial apoptosis pathway. These disruptions result in increased cytoplasmic levels of Cyt C, apoptosis-inducing factor, endonuclease G, and apoptotic protease-activating factor 1, culminating in apoptosis [51]. Furthermore, copper stabilizes hypoxia-inducible factor-1 (HIF-1) and enhances the expression of angiogenic factors. Excessive CuO nanoparticle exposure disrupts mitochondrial dynamics in vascular ECs (VECs) via oxidative stress, leading to increased mitochondrial fission and debris accumulation. This exacerbates ROS production and Cu^+^-induced VEC death [52]. Additionally, a separate study demonstrated that CuO nanoparticles induce oxidative stress through ROS, subsequently activating the endoplasmic reticulum stress pathway. This activation triggers CHOP and caspase-12 apoptosis pathways, ultimately causing the death of rat hepatocytes [53].

After excessive Cu exposure, caspase activation leads to the hydrolysis of deoxyribonuclease inhibitors and subsequent DNA fragmentation. Excess Cu disrupts mitochondrial membrane integrity, resulting in the release of mitochondrial outer membrane proteins (MOMPs) and Cyt C into the cytoplasm. Additionally, Bax—a key factor in the mitochondrial apoptosis pathway—is upregulated in copper-exposed cells. This alters mitochondrial membrane potential, increases membrane permeability, and promotes the continuous release of apoptotic factors [54, 55]. Persistent ROS exposure causes DNA double-strand breaks and base oxidation, triggering a DNA damage response and halting the cell cycle [56]. Rochford et al. demonstrated that Cu(II) complexes induce oxidative stress, leading to DNA damage and the upregulation of heme oxygenase (HO) expression levels. Oxidative stress also fragments mitochondrial structures and increases Cyt C release. Furthermore, mitochondrial regulatory functions become disordered, with upregulated expression of DRP1 and LON protease contributing to mitochondrial fragmentation and the oxidative hydrolysis of misfolded proteins [57]. Overall, these findings demonstrate that Cu and its complexes induce oxidative stress, disrupt mitochondrial structure and function, and trigger cuproptosis.

#### Copper-induced acylation of protein

Copper functions as a cofactor in enzymatic reactions and is critical for the proper folding and conformation of proteins. However, excessive copper ions can disrupt protein conformation and impair their function. Interestingly, ATP production plays an essential role in copper-induced cell death. Research by Tsvetkov et al. demonstrated that copper ion carriers do not significantly impair ATP production in the respiratory chain. This finding suggests that copper does not directly target the electron transport chain but instead impacts components of the TCA cycle [12]. Notably, four enzymes regulate fatty acylation at the entry point of the TCA cycle, and their functions depend on acylation modifications [58]. Mitochondria are the primary site of copper-induced cell death, with key regulators of cuproptosis, including FDX1, LIAS, DLAT, and LIPT1. FDX1 is a critical regulator of copper ionophore-induced cell death. Knocking out FDX1 eliminates protein fatty acylation, conferring resistance to copper-induced cell death [59]. Similarly, LIAS—an enzyme involved in protein fatty acylation metabolism—reduces the toxic effects of cuproptosis when deleted, highlighting its role as a positive regulator of this process [60]. Combined deletion of FDX1 or LIAS, along with copper ion carrier treatment, leads to iron-sulfur cluster protein loss and triggers protein toxicity stress [59]. DLAT, a fatty acylated protein, plays a role in forming the pyruvate dehydrogenase complex via fatty acylation. Binding of Cu^2^^+^ to DLAT reduces iron–sulfur cluster protein expression, increases Hsp70 member 1B expression, and worsens copper toxicity through protein oligomerization. Additionally, FDX1 enhances DLAT lipid acylation, further amplifying copper-induced cytotoxicity [61]. In FDX1 knockout cells, thiooctyl acylation of DLAT and DLST is reduced, leading to increased resistance to copper toxicity. Furthermore, in the absence of FDX1, DLAT and DLST lose their ability to bind copper [12]. This highlights FDX1’s regulatory effects on both DLAT and DLST. Physiological supplementation with copper chloride (CuCl_2_) significantly increases cell death in human rhabdomyosarcoma cells—a phenomenon that is not reversed by other cell death inhibitors [12]. Collectively, these findings reveal that copper promotes fatty acylation and oligomerization of TCA cycle proteins, ultimately driving cell death through the downregulation of iron–sulfur cluster proteins and induction of protein toxicity stress.

#### Copper homeostasis destroys

Copper is a redox-active metal, and its deficiency reduces copper-dependent antioxidant enzymes, leading to the accumulation of ROS and oxidative stress. Therefore, maintaining copper homeostasis is essential for normal physiological functions [62, 63]. Copper homeostasis is tightly regulated by protein transporters and chaperones to ensure proper distribution and to prevent harmful redox reactions [64]. Disruptions in copper homeostasis—caused by genetic mutations, aging, environmental factors, or exogenous substances—disturb copper metabolism. Excess copper in cells activates the transcription factor ACE1, which binds Cu^+^ to regulate copper homeostasis [65]. Detoxification proteins, such as metallothionein and SOD1, are upregulated to neutralize Cu^+^ and scavenge free radicals, preserving SOD1 activity in response to DNA damage [66]. However, excessive Cu^+^ accumulation affects the transcription factor MAC1, leading to DNA strand breaks, base oxidation, and impaired expression of copper transporter genes, which ultimately contribute to cell death [67]. Additionally, the X-linked inhibitor of apoptosis (XIAP) promotes ubiquitination and degradation of the copper metabolism-related protein COMMD1, thereby regulating intracellular copper export. Copper binding to specific cysteine residues in XIAP alters its conformation, accelerates its degradation, and weakens its caspase-inhibiting ability, thereby lowering the apoptosis threshold [68]. Key regulators of copper homeostasis in mammals include CP and CTR1. CP, CTR1, and other copper-related transporters serve as primary carriers for copper exchange in plasma. They facilitate the delivery of copper to cytoplasmic metallochaperones (e.g., ATOX1), mitochondrial metallochaperones (e.g., CCS, SCO1, SCO2, COX11, and COX17), and copper-dependent ATPases (ATP7A and ATP7B), which are involved in copper export and intracellular regulation [69]. Increasing CTR1 expression enhances copper uptake, with CTR1 remaining stable at low copper concentrations but becoming unstable at high concentrations [70]. At elevated Cu^+^ levels, reduced SCO1 synthesis decreases CTR1 abundance, downregulates proteins involved in copper uptake, limits Cu^+^ influx, and mitigates cytotoxicity [71]. Thus, cells require sufficient copper ions to support physiological processes while avoiding excessive copper intake to prevent cytotoxicity and cell death.

#### Cell death induced by copper ionophore

Accumulated copper ions in cells promote the abnormal oligomerization of thiooctyl acylated proteins in the TCA cycle and reduce iron–sulfur cluster protein levels. Copper ion carriers facilitate the transport of copper into cells, thereby increasing intracellular Cu^2^^+^ levels, generating ROS, inhibiting proteasome activity, and inducing tumor cell death. Research indicates that while ES alone does not affect cell growth, co-treatment with Cu^2^^+^ enables ES to transport copper to mitochondria as ES-Cu^2^^+^, inducing cuproptosis via mitochondrial protein toxicity stress mediated by FDX1 [72]. Additionally, ES reduces Cu^2^^+^ to Cu^+^, producing significant ROS, inducing oxidative stress in glioblastoma stem-like cells, and triggering apoptosis [2]. Another study revealed that the copper ion carrier disulfiram reacts with Cu^+^ transported by CTR1 in tumor cells, promoting nuclear accumulation and aggregation of nucleoprotein localization protein 4. This reaction inhibits the ubiquitin-proteasome system, induces endoplasmic reticulum stress, decreases mitochondrial membrane potential, and causes oxidative stress, DNA damage, and the accumulation of cytotoxic protein aggregates [73]. This leads to decreased mitochondrial membrane potential, oxidative stress, DNA damage, and accumulation of cytotoxic protein aggregates [74]. Furthermore, disulfiram suppresses the NF-κB pathway, aldehyde dehydrogenase activity, and T-AOC levels, ultimately leading to cell cycle arrest and apoptosis [75]. He et al. [76] demonstrated that CuONPs induce oxidative DNA damage and cell death through copper ion-mediated p38 MAPK activation in HUVECs, highlighting copper ion release as an upstream activator of CuONP-induced vascular endothelial toxicity. Copper-bound NSC319726 has also been shown to induce ROS production and DNA consumption, culminating in cell cycle arrest [77]. In conclusion, cuproptosis is a newly identified form of cell death closely associated with oxidative stress and mitochondrial respiration. Copper ions directly interact with fatty acyl components in the TCA cycle, leading to oligomerization of fatty acyl proteins, loss of iron–sulfur cluster proteins, and protein toxic stress, ultimately resulting in cell death. The specific pathways of cuproptosis mediated by copper ions include: (a) DNA damage and lipid peroxidation caused by the Fenton reaction; (b) Disruption of mitochondrial pathways, leading to redox imbalance; (c) Interference with protein conformation and function, inhibiting the synthesis of iron–sulfur cluster proteins; (d) Disruption of copper homeostasis, impairing the expression of copper transporters.

## The potential mechanism of AS caused by cuproptosis

**Figure 2.**
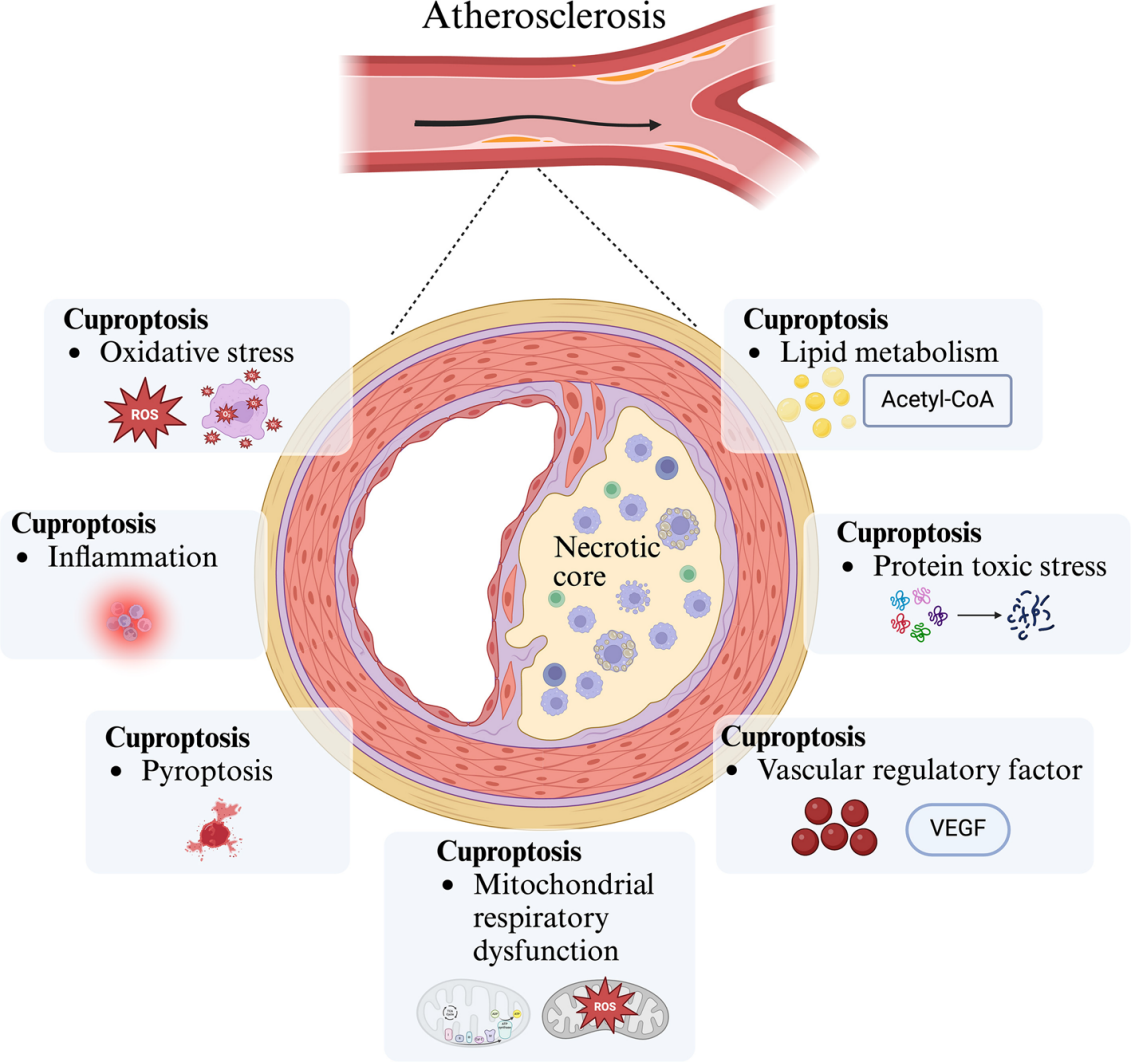
**Possible mechanism of cuproptosis participating in the occurrence and development of AS.** Cuproptosis may promote atherosclerotic plaque formation and lipid protein deposition by regulating oxidative stress, inflammation, pyroptosis, protein toxicity stress, mitochondrial respiratory dysfunction, vascular regulatory factors, lipid metabolism and other ways, and then promote the occurrence and development of AS. AS: Atherosclerosis.

A prospective cohort study reported that elevated serum Cu^+^ levels are associated with the development of AS [78]. Serum Cu^+^ facilitates the oxidation of low-density lipoprotein (LDL) into copper-oxidized LDL, which binds to oxidized LDL receptor 1 on ECs. This interaction upregulates adhesion molecule expression on endothelial surfaces, promoting atherosclerotic plaque formation and progression [79]. Wang et al. [80] observed that plasma copper levels in AS patients increased with the severity of hypercholesterolemia. Similarly, Li et al. demonstrated that copper supplementation in atherosclerotic lesions raised copper concentrations while significantly reducing cholesterol and phospholipid levels, leading to smaller lesions. Plasma copper levels also positively correlate with subclinical carotid plaque formation, identifying copper excess as a novel risk factor for carotid atherosclerotic plaques [81, 82]. These findings suggest a strong link between serum copper levels and AS development, offering new opportunities for CVD prevention and treatment. We hypothesize that cuproptosis is intricately connected to the onset and progression of AS. However, the mechanisms by which cuproptosis influences AS remain unclear. Potential mechanisms (Figure 2) may include oxidative stress, inflammation, pyroptosis, protein toxicity stress, mitochondrial dysfunction, vascular endothelial regulation, and lipid metabolism.

### Oxidative stress

Excessive activation of oxidases, leading to ROS levels surpassing the scavenging capacity of the innate antioxidant defense system, triggers oxidative stress and causes tissue damage [83]. Oxidative stress is a critical pathological factor at all stages of AS, contributing to the oxidative modification of lipoproteins and phospholipids, EC activation, and macrophage foam cell formation [84]. Elevated serum copper levels exacerbate this process, as copper-induced oxidative stress activates inflammatory factors and triggers vascular endothelial inflammation. Copper, as a redox-active metal, enhances ROS production when its levels fluctuate, promoting oxidative stress [85]. Excess intracellular free copper generates ROS through the Fenton reaction, impairing mitochondrial electron transport chain function and leading to excessive ROS formation, hydroxyl radical production, and weakened cellular antioxidant defenses [86, 87]. Copper overload also depletes glutathione (GSH), exacerbating ROS cytotoxicity and oxidative stress [88]. Moreover, it disrupts the expression and activity of antioxidant enzymes, such as superoxide dismutase (SOD), catalase (CAT), and GSH peroxidase (GSH-Px), further increasing ROS production and impairing antioxidant function [51]. In summary, copper overload is a significant driver of oxidative stress, which perpetuates the initiation and progression of AS by activating inflammatory factors. For instance, LDL trapped beneath the endothelium oxidizes into ox-LDL, inducing endothelial dysfunction and promoting atherosclerotic lesion progression [89]. Therefore, oxidative stress represents a central mechanism linking excessive copper to AS development.

### Inflammation

Inflammation is a major research focus and a potential therapeutic target for AS. In 1999, Ross proposed that “atherosclerosis is an inflammatory disease” [90]. During pathological inflammation, tissue copper levels significantly increase [91]. Copper overload has been shown to induce various inflammatory vascular diseases [92]. Specifically, copper overload triggers inflammatory activation in BV2 cells, elevates ROS levels and activating the NF-κB signaling pathway, thereby promoting inflammation in these cells [93]. In the acidic lysosomal environment, CuONPs release copper ions, which disrupt lysosomes and lead to the release of CTSB. CTSB directly mediates the activation of NLRP3 inflammasomes [45]. Additionally, exposure to CuONPs activates the MyD88/TLR4/NF-κB cascade, inducing the expression of pro-IL-1β—an essential step in NLRP3 inflammasomeactivation [94]. Interestingly, tetrathiomolybdate (TTM) significantly reduces serum VCAM1 and ICAM1 levels in ApoE-/- mice and decreases the mRNA expression of adhesion molecules, MCP1, and pro-inflammatory cytokines in the aorta and heart [95]. These findings suggest that TTM mitigates vascular inflammation and slows AS progression by chelating copper and reducing inflammatory mediator expression in vascular cells [96]. The evidence above underscores the critical role of copper overload in driving inflammation, which has been shown to contribute significantly to the initiation and progression of AS [97]. Pro-inflammatory cytokines are particularly crucial in the early stages of AS [98]. In advanced stages, an influx of inflammatory cytokines into the vascular wall degrades collagen fibers in the plaque extracellular matrix, resulting in plaque rupture, hemorrhage, and thrombosis [99]. Moving forward, VECs could serve as an important model for studying how copper overload induces AS lesions through inflammation. Such research may yield valuable insights for developing new AS treatments.

### Pyroptosis

Ankylosing spondylitis is considered a chronic aseptic inflammatory condition. Pyroptosis not only initiates inflammation [100] but also amplifies the inflammatory response through cascade reactions [101], affecting all stages of AS progression [102]. Excessive copper treatment in jejunal epithelial cells significantly increases the gene and protein levels associated with pyroptosis, indicating that copper overload can induce pyroptosis [103]. In hepatocytes, copper overload induces the expression of pyroptosis-related markers, including caspase-1, IL-1β, IL-18, and NLRP3 mRNA, along with a significant increase in related protein levels. However, the combination of a ROS inhibitor, such as NAC, with copper significantly reduces the gene and protein levels linked to pyroptosis. These findings suggest that copper overload-induced pyroptosis is mediated by ROS. Pyroptosis is closely tied to the progression of AS. In the early stages, EC death attracts monocytes and other inflammatory cells, facilitating their recruitment across the endothelium. In advanced atherosclerotic lesions, focal macrophage (Mø) death may promote necrotic core formation and plaque instability. Additionally, the death of focal vascular smooth muscle cells (VSMCs) releases proinflammatory factors, exacerbating inflammation, worsening AS, and contributing to plaque instability [100]. Research exploring the relationship between copper overload and pyroptosis remains limited. Future studies could investigate pyroptosis as a potential mechanism linking copper overload to AS. This could lead to novel strategies for preventing and treating AS.

### Protein toxic stress

During Cu^2^^+^ accumulation in cells, FDX-1 encodes a reductase that reduces Cu^2^^+^ to Cu^+^ and catalyzes the protein acylation of DLST, DLAT, and LIAS. Cu^+^ subsequently binds to the fatty acylation site of DLAT, triggering DLAT oligomerization, protein toxicity stress, and ultimately copper-induced cell death. Protein acylation in mammals is currently known to occur in only four enzymes, all of which form metabolic complexes that regulate the carbon entry point of the TCA cycle: dihydrolipoamide branched-chain transacylase E2, glycine cleavage system protein H, DLAT, and DLST [104]. These enzymes require protein acylation to directly or indirectly participate in the mitochondrial TCA cycle and maintain normal mitochondrial metabolism [58]. However, Cu^+^ binding to fatty acylated proteins in the TCA cycle causes protein-dependent oligomerization, mitochondrial dysfunction, protein toxicity stress, and copper-dependent cell death. Protein toxicity stress is associated with significant ROS production in cells, and ROS is a major contributor to the progression of AS. Elevated ROS levels activate the p38 signaling pathway and induce the production of inflammatory factors, such as IL-6 via the NF-κB pathway. These signaling pathways stimulate the synthesis, differentiation, migration, and proliferation of VSMCs, leading to lipid accumulation and further promoting AS [105]. Additionally, ROS accumulation results in vascular VEC dysfunction by inhibiting endothelial nitric oxide synthase (eNOS), thereby reducing nitric oxide (NO) production. The combined effects of oxidative stress and reduced NO production accelerate AS progression [8]. Protein toxicity stress during cuproptosis may therefore contribute to the onset and progression of AS.

### Mitochondrial respiratory dysfunction

Mitochondria are the primary energy source for cells, producing ATP through oxidative phosphorylation and fatty acid metabolism in the respiratory chain, which acts as the “fuel” for most cellular activities [106]. They account for approximately 90% of cellular oxygen consumption and are a critical target of copper toxicity [64]. The cardiovascular system has particularly high energy demands, with mitochondrial respiration fulfilling most of these requirements. During cuproptosis, FDX1 reduces Cu^2^^+^ to Cu^+^, releasing it into the mitochondrial matrix. This process disrupts the synthesis of iron–sulfur cluster proteins, which are essential cofactors for the mitochondrial respiratory chain [107]. Excess copper damages the genes involved in iron–sulfur cluster protein synthesis and impairs mature iron–sulfur cluster-containing metalloenzymes [108–110]. Such disruption compromises the mitochondrial respiratory complex, leading to mitochondrial dysfunction and, ultimately, cell death [111]. Mitochondrial dysfunction is closely associated with the onset and progression of AS [112, 113]. It increases ROS production, which contributes to mtDNA damage, ox-LDL accumulation in vessel walls, and lipid plaque formation on arterial walls [114]. Excess copper may also trigger the opening of mitochondrial permeability transition pores, release pro-apoptotic factors, and induce cell death [115]. Mitochondrial respiratory dysfunction exacerbates atherosclerotic lesion progression by impairing endothelial function, altering VSMC proliferation or apoptosis, and influencing macrophage polarization. In VECs, mitochondrial dysfunction worsens endothelial injury and inflammatory responses during AS progression [116]. Additionally, it promotes lipid accumulation, oxidative stress, inflammation, and VSMC proliferation, all of which drive plaque formation [117, 118]. Given the established role of mitochondrial respiratory dysfunction in the onset and progression of AS, the potential contribution of cuproptosis to AS progression through mitochondrial impairment warrants further investigation.

### Vascular regulatory factor

HIF-1 is a heterodimeric core transcription factor that plays a central role in regulating oxygen homeostasis and is widely expressed in mammalian and human cells [119]. HIF-1α, in particular, is critically involved in the onset and progression of AS. The hypoxic environment within AS plaques induces stable expression of HIF-1α, which exacerbates plaque progression by contributing to endothelial dysfunction, smooth muscle cell proliferation and migration, phenotypic transformation, and immune cell infiltration—ultimately creating a vicious cycle [120]. Copper is a key regulator of HIF-1 activity through several mechanisms, including stabilizing HIF-1α, facilitating the formation of transcription complexes, and enabling HIF-1 binding to the HRE of target genes [121]. The interaction between HIF-1 and HRE drives copper-dependent expression of angiogenesis-related genes, such as VEGF and BNIP3 [122, 123]. Additionally, the CTR1/ATOX1/ATP7A/Rac1 pathway facilitates the conversion of pro-LOX to LOX during copper transport [124]. LOX and LOX-like proteins promote cross-linking between elastin and matrix collagen. Copper deficiency, on the other hand, reduces LOX levels and activity, leading to collagen and elastin fiber degradation as well as vascular endothelial damage [125]. These findings highlight how imbalances in copper homeostasis can disrupt vascular regulatory factors and contribute to the development of AS.

### Lipid metabolism

Copper contributes to the oxidation of LDL particles, and fluctuations in copper levels may influence their susceptibility to oxidative damage. Excess copper promotes LDL oxidation, leading to the production of ox-LDL and accelerating the progression of AS [126]. Furthermore, copper is involved in lipid metabolism, including fatty acid synthesis, cholesterol synthesis, and lipoprotein metabolism. Research indicates that serum copper concentrations are positively correlated with increased serum total cholesterol and HDL levels, as well as a heightened risk of dyslipidemia associated with elevated total cholesterol and HDL levels [127]. Another study reported a positive correlation between serum copper levels and fasting serum triglyceride concentrations in CHD patients diagnosed via angiography [128]. Animal studies have shown that excessive copper intake disrupts myocardial lipid metabolism, significantly raising serum cholesterol, LDL cholesterol, and aspartate aminotransferase levels. These disruptions in glycerophospholipid metabolism and fatty acid degradation pathways contribute to myocardial injury [129]. In zebrafish, excessive copper intake elevated liver copper content and upregulated genes involved in lipogenesis. Notably, it increased the mRNA levels of LXRα and SREBP-1 signaling pathways, mediating triglyceride synthesis and accumulation through transcriptional regulation of lipogenesis and lipid storage genes [130]. Similarly, experimental studies on grass carp revealed that elevated waterborne copper levels caused lipid accumulation in the liver. Triglyceride content and lipogenic enzyme activity were significantly upregulated. Excess copper inhibited the Wnt/β-catenin pathway, preventing β-catenin nuclear accumulation, downregulating sirt1 mRNA and protein expression, upregulating β-catenin acetylation, and promoting lipogenesis and lipid deposition. These findings collectively highlight how excessive copper intake disrupts lipid metabolism and contributes to AS progression.

## Clinical application of cuproptosis targeting strategy in AS

In clinical practice, maintaining copper balance is critical, as identifying biomarkers that reflect changes in copper metabolism is essential for detecting AS risk early, before clinical symptoms emerge. This proactive approach facilitates timely interventions and improves patient prognosis. Alterations in copper homeostasis observed in atherosclerotic diseases, alongside the discovery of cuproptosis-related genes, reveal promising therapeutic targets [131]. Researchers have identified five cuproptosis-related genes—F5, MT4, RNF7, S100A12, and SORD—as potential biomarkers for CHD diagnosis [132]. Furthermore, in 2023, Yuting Cui’s team became the first to report the critical roles of cuproptosis-related genes FDX1, SLC31A1, and GLS in AS formation. Their findings showed that FDX1 and SLC31A1 expression were upregulated in atherosclerotic plaques, while GLS expression was downregulated [10]. The identification of these biomarkers underscores the significant role of cuproptosis in AS progression. Given cuproptosis’s impact on AS onset and progression, restoring copper homeostasis emerges as a promising therapeutic target for AS treatment.

### Potential biomarkers for diagnosing AS

#### Ferredoxin 1 (FDX1)

Ferredoxin is a key regulator of cuproptosis, directly binding to copper and activating proteotoxic stress [46]. Tsvetkov et al. [12] demonstrated that FDX1 reduces Cu^2^^+^ to Cu^+^. Knocking out the FDX1 gene provides cells with greater resistance to copper-induced cell death. Notably, cells can be protected from copper toxicity by knocking out either FDX1 or lipase-related enzymes, suggesting that FDX1 acts as an upstream regulator of protein acylation. Another study revealed that FDX1 deficiency impairs ATP production, particularly in glucose metabolism, fatty acid oxidation, and amino acid metabolism [133]. FDX1 regulates mitochondrial protein acylation upstream, with lipoic acid serving as a critical substrate for this process. Joshi further demonstrated that FDX1 is essential for producing the lipoic acid cofactor and may contribute to AS formation by upregulating endogenous lipoic acid expression [134].

#### Solute carrier family 31 member 1 (SLC31A1)

The copper transporter solute carrier family 31 member 1 plays a critical role in importing copper from circulation and maintaining copper homeostasis in most cells [135]. Functioning as a key transporter responsible for copper uptake on the cell membrane, SLC31A1 has been identified as a significant factor in inducing cuproptosis when overexpressed [134]. Das et al. [136] demonstrated that SLC31A1 not only senses ROS but also promotes angiogenesis. In AS, the accumulation of macrophages is strongly associated with plaque destabilization and rupture, which can result in acute atherothrombotic events [137]. Notably, SLC31A1 is specifically upregulated in advanced or unstable plaques enriched with macrophages. This suggests that SLC31A1 may induce macrophage cuproptosis, thereby contributing to plaque instability [10].

#### Glutaminase (GLS)

Glutaminase (GLS), an amide hydrolase, catalyzes the conversion of glutamine into glutamic acid and ammonium ions [138, 139]. Primarily localized in mitochondria [140], GLS plays a key role in converting glutamine into glutamate, which supports the synthesis of intracellular GSH. This, in turn, mitigates ROS-induced damage and protects cells from copper toxicity [141, 142]. Recent studies have shown that GLS transcripts are downregulated in human plaque tissue compared to normal vessels. Double-fluorescence labeling has further confirmed GLS localization in VSMCs. These findings indicate that excessive copper disrupts the GLS-driven redox system in VSMCs, exacerbating copper toxicity and contributing to the progression of AS.

### Potential therapeutic strategies for cuproptosis-induced AS

#### Copper chelator

Copper chelating agents can non-selectively lower intracellular Cu^+^ concentrations, thereby inhibiting cuproptosis. Several copper chelators have shown potential in animal studies and clinical trials for treating AS [143]. TTM is a highly specific copper chelating agent that reduces bioavailable copper levels and vascular inflammation, thereby inhibiting the development of AS lesions. Studies demonstrated that TTM administration significantly reduced copper levels in the aorta of ApoE-/- mice, indicating its role in slowing AS progression by lowering bioavailable copper [96]. Furthermore, TTM mitigates TNF-α-induced activation of NF-κB and AP-1, as well as the mRNA and protein expression of VCAM-1, ICAM-1, and MCP-1. This evidence suggests that TTM reduces TNF-α-induced EC activation, thereby contributing to the inhibition of AS progression [144]. Given the long-term effects of metal and natural chelating agents, additional research is required to fully explore their clinical applications.

#### Copper ionophore

Copper ionophores selectively transport and release copper ions by binding to Cu^+^, addressing intracellular copper deficiency. This targeted transport reduces non-specific damage and enhances treatment efficacy. For instance, Elisimo, a copper ion carrier with tumor cell selectivity, targets mitochondrial metabolism to induce cuproptosis [145]. Similarly, 8-hydroxyquinoline, another copper ion carrier, facilitates the reduction of Cu^2^^+^ to Cu^+^ for intracellular transport and chelation [146]. However, conventional copper ion carriers often lack specificity and exhibit multifunctionality, which can lead to unintended cellular damage. To address this issue, multifunctional nanocomposites have been developed to enable precise delivery of drugs and molecules, thereby increasing specificity and targeting efficiency. Copper ionophore-mediated cell death, strongly linked to mitochondrial metabolism, is closely associated with AS progression. These findings suggest that copper ionophores hold promise as a novel therapeutic strategy for targeting cuproptosis in AS.

#### Copper chaperone small molecule inhibitor

Copper ion chelating agents are known to reduce intracellular Cu^+^ levels, but their non-specific chelation of other metals often leads to severe toxicity and side effects. Consequently, alternative approaches that minimize side effects while enabling precise control of copper concentrations are needed. Copper chaperone proteins play a critical role in the process of cuproptosis. For instance, the compound DCAC50 inhibits Cu^+^ binding and transport by copper chaperones ATOX1 and CCS, selectively suppressing cancer cell proliferation without significantly impacting normal cell survival [147]. Additionally, the copper chaperone protein ATOX1 has been implicated in neointimal formation after vascular injury, as it facilitates VSMC migration and the recruitment of inflammatory cells to the injury site. These findings highlight ATOX1 as a promising therapeutic target for AS [147].

#### Copper homeostasis nano-regulators

The development of nanomedicines designed to maintain copper homeostasis demonstrates significant potential for treating AS [131]. Turmeric, a natural metal-ion chelating agent, contains curcumin as its primary active component. Curcumin scavenges free radicals and helps preserve antioxidant enzyme levels in the presence of copper. Recent studies emphasize the promise of targeted delivery techniques for curcumin in AS treatment [148]. Additionally, dual-catalyst CuTPP/TiO_2_ nanoparticles exhibit antithrombotic, regulatory, and anti-inflammatory effects on vascular wall cells, including endothelial and smooth muscle cells. These nanoparticles function by releasing NO signaling molecules and eliminating harmful ROS, thereby slowing the progression of AS [149].

## Conclusion

Artery disease ranks as the 11th leading cause of mortality and disability among countries within the Organization for Economic Cooperation and Development (OECD) [150]. Although arterial disease accounted for only 1.22% of all cardiovascular-related fatalities in the United States in 2021, its growing burden highlights a critical area for public health intervention [151]. CVD remains the leading cause of death and disability among women worldwide [152]. AAS serves as the common pathological foundation for cardiovascular and cerebrovascular diseases, including CHD, myocardial infarction, and stroke. Therefore, understanding the pathological process of AS is essential for developing effective cardiovascular protection strategies. Copper is a vital trace element necessary for growth, development, and functioning as a cofactor for numerous enzymes. Recent research has illuminated the correlation between abnormal copper ion metabolism and AS. This paper reviews several mechanisms through which cuproptosis, a copper-dependent form of regulated cell death, may contribute to AS. These mechanisms include inducing oxidative stress, promoting inflammation, triggering cell scorching, and facilitating copper-dependent cell death, all of which affect the formation and rupture of atherosclerotic plaques. However, the pathological mechanisms underlying AS are not yet fully understood. Cuproptosis is closely tied to cardiovascular pathology, primarily as a driver of ROS excess. It is also associated with protein toxic stress and mitochondrial respiratory dysfunction. Further research is necessary to elucidate the interactions among these factors and their role in AS progression. Modern studies have identified active ingredients that may help prevent AS by inhibiting copper overload in the cardiovascular system. Furthermore, cuproptosis-related genes, such as FDX1, SLC31A1, and GLS show potential for diagnosing AS. Restoring copper homeostasis and inhibiting cuproptosis represent promising strategies for treating AS. In conclusion, cuproptosis plays a significant role in AS pathogenesis. Its inhibition could offer a novel approach to both diagnosing and treating AS, paving the way for more effective cardiovascular care.

Cuproptosis has deepened our understanding of copper’s role in AS. The link between cuproptosis, mitochondrial dysfunction, and AS underscores the need to further investigate these molecular mechanisms. However, the absence of specific biomarkers for cuproptosis poses a major challenge to its clinical application. Future research should focus on identifying potential biomarkers of cuproptosis on a large scale using advanced quantitative techniques, such as gene chips, proteomics, and metabolomics. These biomarkers should then be validated for specificity, sensitivity, and stability using clinical samples to establish their diagnostic utility. In parallel, developing animal models related to cuproptosis will be crucial. Such models can simulate the onset and progression of human diseases, offering an essential platform to study the pathophysiological mechanisms of cuproptosis and evaluate potential therapeutic effects. Animal models will also help verify the *in vivo* reliability and validity of identified biomarkers. While recent studies have provided valuable insights into cuproptosis, several critical questions remain unanswered: Can cuproptosis serve as a therapeutic target for AS? What are the precise mechanisms by which cuproptosis mediates AS? Furthermore, which compounds—including traditional Chinese medicines and their active components—can mitigate AS triggered by cuproptosis? Addressing these challenges will pave the way for novel AS prevention and treatment strategies, providing a solid scientific foundation for future therapies.
